# Safety of the methylene blue plus chloroquine combination in the treatment of uncomplicated falciparum malaria in young children of Burkina Faso [ISRCTN27290841]

**DOI:** 10.1186/1475-2875-4-45

**Published:** 2005-09-22

**Authors:** Peter E Meissner, Germain Mandi, Steffen Witte, Boubacar Coulibaly, Ulrich Mansmann, Jens Rengelshausen, Wolfgang Schiek, Albrecht Jahn, Mamadou Sanon, Théophile Tapsoba, Ingeborg Walter-Sack, Gerd Mikus, Jürgen Burhenne, Klaus-Dieter Riedel, Heiner Schirmer, Bocar Kouyaté, Olaf Müller

**Affiliations:** 1 Department of Tropical Hygiene and Public Health, Ruprecht-Karls-University, INF 324, 69120 Heidelberg,, Germany; 2 Department of Paediatrics IV Neonatology, Ruprecht-Karls-University, Heidelberg, Germany; 3 Centre de Recherche en Santé de Nouna, Burkina Faso, POB 02, Nouna, Burkina Faso; 4 Institute of Medical Biometrics and Informatics, Ruprecht-Karls-University, Heidelberg, Germany; 5 Institute of Medical Information, Biometry, and Epidemiology, Ludwig-Maximilians-University, Munich, Germany; 6 Department of Internal Medicine VI, Clinical Pharmacology and Pharmacoepidemiology, Ruprecht-Karls University, Heidelberg, Germany; 7 DSM Fine Chemicals, Linz, Austria; 8 Biochemistry Center, Ruprecht-Karls-University, Heidelberg, Germany

## Abstract

**Background:**

Safe, effective and affordable drug combinations against falciparum malaria are urgently needed for the poor populations in malaria endemic countries. Methylene blue (MB) combined with chloroquine (CQ) has been considered as one promising new regimen.

**Objectives:**

The primary objective of this study was to evaluate the safety of CQ-MB in African children with uncomplicated falciparum malaria. Secondary objectives were to assess the efficacy and the acceptance of CQ-MB in a rural population of West Africa.

**Methods:**

In this hospital-based randomized controlled trial, 226 children (6–59 months) with uncomplicated falciparum malaria were treated in Burkina Faso. The children were 4:1 randomized to CQ-MB (n = 181; 25 mg/kg CQ and 12 mg/kg MB over three days) or CQ (n = 45; 25 mg/kg over three days) respectively. The primary outcome was the incidence of severe haemolysis or other serious adverse events (SAEs). Efficacy outcomes were defined according to the WHO 2003 classification system. Patients were hospitalized for four days and followed up until day 14.

**Results:**

No differences in the incidence of SAEs and other adverse events were observed between children treated with CQ-MB (including 24 cases of G6PD deficiency) compared to children treated with CQ. There was no case of severe haemolysis and also no significant difference in mean haemoglobin between study groups. Treatment failure rates were 53.7% (95% CI [37.4%; 69.3%]) in the CQ group compared to 44.0% (95% CI [36.3%; 51.9%]) in the CQ-MB group.

**Conclusion:**

MB is safe for the treatment of uncomplicated falciparum malaria, even in G6PD deficient African children. However, the efficacy of the CQ-MB combination has not been sufficient at the MB dose used in this study. Future studies need to assess the efficacy of MB at higher doses and in combination with appropriate partner drugs.

## Introduction

Malaria is globally responsible for 1.5 – 2.7 million deaths per year and remains a leading cause of mortality in children less than five years of age, especially in African countries [38,5]. Only a few safe and effective antimalarial drugs are presently affordable for the great majority of sub-Saharan African (SSA) populations [4,40]. The increasing resistance of *Plasmodium falciparum *to chloroquine and other well-known antimalarials like sulphadoxine/pyrimethamine demonstrates the urgency of developing new drugs against malaria [36]. The combination of the drugs chloroquine (CQ) and methylene blue (MB) appears to be a promising new regimen in regions where CQ sensitivity prevails [32].

MB is a registered drug for the treatment of methaemoglobinemia and is also applied for cancer treatment, mainly at i.v. dosages of 1–2 mg/kg [18]. MB was already successfully used over 100 years ago for the treatment of malaria, even in children [14,9,11]. It was forgotten when other drugs (e.g. chloroquine) were introduced to the market. In recent years *in vitro *experiments have confirmed the high antimalarial potency of MB [1]. MB specifically inhibits the glutathione reductase of the malarial parasite and it prevents the polymerization of haem into haemozoin similar to 4-amino-quinoline antimalarials [31,32]. It has, thus, the potential to reverse CQ resistance [10,22,13].

There is some concern that the application of MB could be followed by haemolysis in glucose-6-phosphate dehydrogenase (G6PD) deficient individuals in malaria-endemic regions. This is likely to be not clinically relevant in West Africa where the milder form (class III) of G6PD deficiency dominates [12]. Moreover, the safety of CQ-MB is supported by the results of two studies in adult populations in 2003. The combination was well tolerated in healthy G6PD sufficient adult males and females from Germany and in healthy G6PD deficient adult males from Burkina Faso [30,20].

The primary objective of the present trial was to study the safety of CQ-MB in young West African children with uncomplicated falciparum malaria. Secondary objectives were to assess efficacy and acceptance of the combination.

## Materials and methods

### Study area and patients

The study was conducted in the district hospital of Nouna in the province of Kossi in north-western Burkina Faso. Nouna town has about 20,000 inhabitants of different ethnic groups. Formal health services in the Kossi province are restricted to the district hospital and to a small number of village-based health centres. Therefore, access to health services is limited, particularly during the rainy season [26]. The area is highly endemic for *P. falciparum *malaria [25]. Most malaria transmission and disease takes place during or shortly after the rainy season, which lasts from July-October. CQ is the first-line treatment for uncomplicated malaria in Burkina Faso. Day 14 CQ clinical failure rates in the villages surrounding Nouna town were shown to be around 10% in recent years [27].

The study was explained to the population of Nouna town during a series of community meetings prior to selection of study children. In October and November 2003, consecutive children aged 6–59 months presenting with uncomplicated falciparum malaria (axillary temperature ≥ 37.5°C and ≥ 2,000 *P. falciparum *asexual parasites per μl blood) at the outpatient department of Nouna district hospital and whose parents or caretaker had given informed consent were enrolled in the study. Most study children were from the town of Nouna, only a few children were from villages close to Nouna. Children with complicated or severe malaria (repeated vomiting, seizures or other neurological impairment), anaemia (haemoglobin <8 g/dl or haematocrit <24%) or any other apparent significant disease (e.g. pneumonia, meningitis, hepatitis, severe diarrhoea, measles, severe malnutrition) were excluded from the trial.

### Study design and procedures

The study was designed as a sex-stratified (male:female, 4:1) parallel group randomized controlled trial (RCT). In each sex stratum, children were block-randomized by envelopes to receive either treatment with CQ or CQ-MB by computer-generated, randomly permutated codes. The randomization ratio of CQ-MB : CQ was 4:1. The unbalanced stratification and randomization scheme was justified by the need to achieve a high number of G6PD deficient children treated with CQ-MB. The prevalence of G6PD deficiency in boys under five years is 18%, while the prevalence in girls is below 4% [7]. The study was open label, with blinding only for the laboratory technicians responsible for parasite determination in blood smears.

All study children received a standard total dose of 25 mg/kg of CQ syrup (10 mg/ml) over a period of three days (first and second day: 10 mg/kg, third day: 5 mg/kg). Chloroquine was taken from the quality-controlled essential drug stock of the Ministry of Health. The CQ-MB group additionally received orally a 0.5% MB solution (4 mg/kg/day) for three days, divided into morning and evening doses (produced by Mayrhofer Pharmazeutika / Linz, Austria).

All study children were hospitalized for four days, and study medications were given according to weight-based tables by a study nurse, directly supervised by a study physician. Axillary temperatures were taken every six hours during the time of hospitalization. At least once during the time of hospitalization, the colour of the urine was checked to assess MB compliance through visual observation. In case of vomiting within half an hour after the study medication, the medication was repeated. All drugs were allowed as concomitant treatments, except dapsone and other sulfones, acetanilide and phenacetin, nalidixic acid, niridazole, nitrofurantoins and sulphonamides (themselves known to cause haemolysis in certain types of inherited G6PD deficiency) [12]. All children having fever ≥ 38.5°C received standard doses of paracetamol (10 mg/kg every six hours) until symptoms subsided.

Study participants were examined by the study physicians twice daily until discharge from hospital and again after 14 days. Treatment failures were treated according to national guidelines with standard doses of sulfadoxine-pyrimethamine (currently still the second-line treatment in Burkina Faso) or with quinine i.m. or i.v. if indicated. Acceptance of MB was evaluated at day 14 by asking the caretakers standardized questions about adverse effects.

At inclusion, every day in hospital and on day 14 a blood sample was taken and malaria parasitaemia (thin and thick blood smears) as well as haemoglobin (in case of venous blood sampling) and haematocrit values were measured. After Giemsa-staining, all blood smears were examined by two experienced laboratory technicians blinded to the group allocation. In case of disagreement, a third laboratory technician examined the slides. Thick and thin blood films were analysed for the species-specific asexual parasite density per μl by counting against 200 white blood cells and multiplying by 50. Slides were declared negative if no parasites were seen in 400 fields on the thick film. Re-examination of a 10% random sample of blood films at the laboratory of the Heidelberg School of Tropical Medicine showed a 95% concordance of malaria diagnoses. Furthermore, serum creatinine levels were monitored with a spectrophotometer (Ultrospec 1000^®^) during the time of hospitalization.

The G6PD status was determined at inclusion using the NADPH fluorescence test of Beutler and Mitchell [2] in miniaturized form on paper (NFP-test) [6,7]. The G6PD results were later validated by PCR. Sequences flanking the putative mutations were amplified in two separate nested PCR assays [17]. The alleles were distinguished by restriction fragment length polymorphism. Finally, genetic resistance to CQ in the *P. falciparum *isolates was estimated by analysing the prevalence of the mutation K76T in the *Pfcrt *gene product according to Djimde *et al. *[8]. Both, G6PD PCR and CQ resistance marker diagnosis were conducted at the Tropical Institute in Berlin (Germany).

The primary study endpoint was safety, assessed by the frequency and proportion of children with at least one serious adverse event that could be possibly, probably or definitely related to the drug. Secondary endpoints were efficacy outcomes, judged by the incidence of early treatment failures (ETF) on day four and late clinical failures (LCF), clinical failure (CF = ETF+LCF) and late parasitological failures (LPF) on day 14; fever clearance time, parasite clearance time, change in haematocrit after four days compared to baseline, incidence of observed or self-reported adverse events over the 14 days follow-up period and monitoring of concomitant drug intake. In addition, the impact of G6PD status based on the NFP-test and PCR genotyping on safety parameters was investigated. An adverse event was defined according to internationally established principles for Good Clinical Practice (GCP).

### Statistical methods

With a study population of 200 children, 160 were randomized into the CQ-MB group and with high probability (80%) at least 22 G6PD deficient children will be exposed to MB. In order to reject the Null hypothesis of a high haemolysis risk (>20%) on a significance level of 5% and with a power of 90% if the true risk is small (<2%), 22 G6PD deficient children were to be included. A total of 225 patients was planned and a 10% drop-out rate was assumed. The WHO definitions for ETF, LCF and LPF were applied [39]. Study days were defined as 24 h intervals after first drug intake. Losses to follow-up and drop-outs due to other reasons were considered as ETF or LCF, depending on the time of drop-out in an intention-to-treat manner. All tests used apart from the primary analysis have exploratory character. The (continuity adjusted) Chi square test (Chi) was used to compare rates, the non-parametric Wilcoxon-Mann-Whitney test (WMW) to compare metric or ordinal data. If possible, estimates and the corresponding 95% confidence interval are given. The statistical calculation was performed with SAS release 8.02 (SAS^® ^Institute Inc, Cary, NC, USA).

Roughly 5% mortality over the first five years of childhood in malaria endemic areas of SSA is directly caused by malaria [34]. This implies an annual malaria specific mortality risk of 1%. From a public health-related risk benefit calculation it follows that a risk for life-threatening haemolysis above 10% is not acceptable for a first-line antimalarial drug, because it could increase the overall childhood mortality. Assuming a G6PD deficiency prevalence of at least 10% in the CRSN study area and the fact that every child in the CRSN study area will be treated at least once per year with a malaria drug which eliminates malaria related mortality, but implies life-threatening haemolysis in less than 10% of G6PD deficient children, the annual population risk would be below 1% (0.1 × 0.1).

### Ethical aspects

The trial was conducted in accordance with local law, the internationally established principles for Good Clinical Practice, which had their origin in the Declaration of Helsinki of the World Medical Association, and in accordance with the "Note for Guidance on Clinical Investigation of Medicinal Products in Children". Data collection and analysis followed established quality principles. The protocol was approved by the Ethics Committee of the Medical Faculty of Heidelberg University and the local Ethics Committee in Burkina Faso. The safety of the trial was also monitored by a data safety monitoring board (DSMB).

After having received detailed information from the study physician about all risks and benefits of the study through translation of a detailed research consent form into the local language, caretakers were asked for their written consent. They were clearly informed that they could withdraw from the study at any time and without disadvantage. A standard blood transfusion service was available at the hospital, and study physicians and emergency medications were available 24 hours per day. For the study participants not only malaria treatment but all treatments were free of charge. Furthermore, all children in the specified age group who were presented to the hospital during the study period for other conditions besides malaria also received free treatment.

## Results

### Study group characteristics

**Figure 1:**
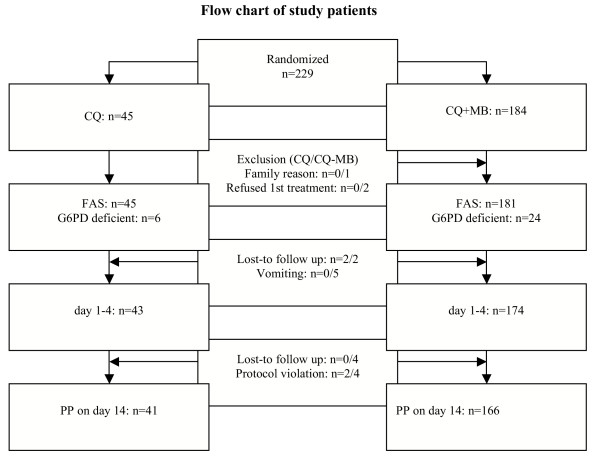
Flow chart of study patients.

229 children with uncomplicated malaria were enrolled (Figure 1). Three boys in group CQ-MB were excluded on day one, one left for family reasons before application of the first medication, two refused to take the first dose of MB. The following analysis is based on the remaining 226 children. This is the full analysis set (FAS) for the intention to treat analysis, 45 receiving CQ and 181 CQ-MB. In group CQ and CQ-MB respectively 41/45 and 166/181 children had taken the study drugs as per protocol (= PP, Figure 1). There were no statistically significant differences in sex ratio, age (p_WMW _= 0.801), weight (p_WMW _= 0.898), creatinine, malaria parasitaemia and the prevalence of the genetic resistance marker *pfcrt *K76T of the malaria parasites (p_Chi _= 0.793) between the two groups (Table 1). There was no difference in the haemoglobin (p_WMW _= 0.864) but in haematocrit (p_WMW _= 0.007) values between the treatment groups at baseline (Table 1). G6PD status was assessed in all 226 children. Overall, 30/226 children (13.3%, 95% CI [9.1%, 18.4%]) were diagnosed as G6PD deficient using the NFP test (six in the CQ group, 24 in the CQ-MB group) (Table 1). There was good agreement between the NFP test and the PCR results; these findings are published separately [23].

**Table 1:** Baseline characteristics by study group

**Characteristic**	**CQ (N = 45)**	**CQ-MB (N = 181)**	**Total (N = 226)**
**Sex**			
- male	36(80.0%)	145(80.1%)	181(80.1%)
- female	9(20.0%)	36(19.9%)	45(19.9%)
**Age [months]**			
- Mean ± SD	30.0 ± 13.1	29.5 ± 14.9	29.6 ± 14.5
- Median	28.0	27.0	27.5
**Weight [kg]**			
- Mean ± SD	10.5 ± 2.8	10.6 ± 2.8	10.6 ± 2.8
- Median	10.0	10.0	10.0
**G6PD deficiency (NFP-test)**			
- No	39 (86.7%)	157 (86.7%)	196 (86.7%)
- Yes	6 (13.3%)	24 (13.3%)	30 (13.3%)
**pfcrt gene**			
- wildtype	11 (24.4%)	40 (22.6%)	51 (23.0%)
- K76T mutant	34 (75.6%)	137 (77.4%)	171 (77.0%)
- Missing data	0	4	4
**Haemoglobin [g/dl]**			
- Mean ± SD	10.3 ± 1.3	10.4 ± 1.4	10.4 ± 1.4
- Median	10.2	10.2	10.2
**Haematocrit [%]**			
- Mean ± SD	27.9 ± 2.6	29.4 ± 3.3	29.1 ± 3.2
- Median	28.0	30.0	28.0
**Creatinine [μmol/l]**			
- Mean ± SD	75.3 ± 15.2	77.2 ± 14.5	76.8 ± 14.6
- Median	74.4	76.4	75.3
***P. falciparum *parasites/μl log10 values**			
- Mean ± SD	4.28 ± 0.54	4.35 ± 0.54	4.33 ± 0.54
- Median	4.32	4.38	4.36
- Min, Max	3.40, 5.29	3.08, 5.71	3.08, 5.71

### Compliance with study drugs

The compliance is illustrated in Figure 1. In some cases, particularly in the CQ-MB group, the drug administration needed repeating after being initially rejected by the children (1/45 CQ, 35/181 CQ-MB). Application of the bitter-tasting 0.5% MB solution was more difficult in children <2 years compared to older children (26/61 versus 9/120, p_Chi _< 0.0001).

### Safety of study drugs

There was no case of severe haemolysis within the group of 24 G6PD deficient children who received MB (one-sided exact 95% CI [0%, 11.73%]). Combining these results with the pilot study in adult males [20], no haemolysis was observed in 98 G6PD-deficient subjects under CQ-MB. This updates the risk estimate to 3% (upper confidence bound). One patient in the G6PD sufficient CQ-MB group had a serious adverse event (SAE; prolonged hospitalization) which was unrelated to the study medication. The haemoglobin of this 21 months old girl with an initially high parasitaemia of 193,000/μl and haemorrhagic diarrhoea dropped within four days from 11.8 g/dl to 6.6 g/dl. There was no need for blood transfusion. With quinine and antibiotics she quickly recovered within five days after admission to hospital. There was only one febrile child with severe jaundice (group CQ-MB), later diagnosed to have acute hepatitis A. There was also no marked difference in the incidence of adverse events between the two groups (Table 2). Only pruritus was observed with a higher incidence in the CQ group compared to the CQ-MB group (8/45 versus 9/181; p_Chi _= 0.017). Until the end of hospitalization there was no significant increase of serum creatinine in the CQ-MB versus the CQ group.

**Table 2:** Adverse events according to MedDRA SOC by study group

	**CQ (N = 45)**	**CQ-MB (N = 181)**
**Gastrointestinal disorders**	23 (38.3%)	93 (41.5%)
**Respiratory disorders**	16 (26.7%)	78 (34.8%)
**Infections and infestations**	10 (16.7%)	38 (17.0%)
**Skin and subcutaneous tissue disorders**	8 (13.3%) ^a^	9 (4.0%) ^a^
**Others**	3 (5.1%)	6 (2.7%)

^a ^p = 0.017

There were no differences between CQ and CQ-MB study groups in haemoglobin and haematocrit values over time in both the G6PD deficient and sufficient subgroups (Table 3). The absolute median increase in haematocrit from baseline until day 14 was 2% with no differences between the overall study groups and between the G6PD deficient subgroups.

**Table 3:** Changes of laboratory values between baseline and study day 3 (haemoglobin, Hb), day 4 (haematocrit, HCT) and day 14 (HCT) by study group and G6PD status.

	**CQ**	**CQ-MB**	**Group Comparison**
**Baseline – day 3/4: G6PD deficient children**			
Changes in Hb [g/dl] (N = 26)	0.4 ± 1.2	-0.7 ± 1.5	[-0.4; 2.5], p_WMW _= 0.126
Changes in HCT [%] (N = 30)	1.3 ± 4.1	-1.4 ± 3.9	[-0.9; 6.4], p_WMW _= 0.259
**G6PD sufficient children**			
Changes in Hb [g/dl] (N = 176)	-0.5 ± 1.3	-0.4 ± 1.6	[-0.6; 0.6], p_WMW _= 0.906
Changes in HCT [%] (N = 193)	-1.2 ± 2.8	-1.1 ± 4.1	[-1.5; 1.3], p_WMW _= 0.871
**Baseline – day 14: G6PD deficient children**			
Changes in HCT [%] (N = 30)	1.3 ± 3.3	1.4 ± 3.4	[-3.2; 3.0], p_WMW _= 0.979
**G6PD sufficient children**			
Changes in HCT [%] (N = 187)	1.9 ± 3.8	1.2 ± 5.1	[-1.1; 2.5], p_WMW _= 0.698

Mean ± SD and 95% CI for the mean difference; no difference in change of Hb, HCT between G6PD deficient and sufficient children in the CQ-MB group for Hb (p_WMW _= 0.538), HCT (day 4, p_WMW _= 0.897) and HCT (day 14, p_WMW _= 0.933)

### Efficacy of study drugs

Among the fully compliant patients there were 22/41 (53.7%, 95% CI [37.4%; 69.3%]) treatment failures in group CQ (15 ETF, 7 LCF) and 73/166 (44.0%, 95% CI [36.3%; 51.9%]) in group CQ-MB (48 ETF, 25 LCF). Comparing CQ and CQ-MB no significant differences were found regarding CF (p_Chi _= 0.348, OR = 0.68, 95% CI [0.34; 1.35]), ETF (p_Chi _= 0.444, OR = 0.71, 95% CI [0.34; 1.45]), LCF (p_Chi _= 0.938, OR = 0.86, 95% CI [0.34; 2.16]) and LPF (p_Chi_= 1.0, OR = 1.04, 95% CI [0.37; 2.95]) (Table 4).

**Table 4:** Efficacy according to study group (per protocol analysis)

	**CQ (N = 41)**	**CQ-MB (N = 163)**
**Clinical failure (CF)**	22 (53.7%)	73 (44.0%)
**Early treatment failure (ETF)**	15 (36.6%)	48 (28.9%)
**Late clinical failure (LCF)**	7 (17.1%)	25 (15.1%)
**Late parasitological failure (LPF)**	5 (12.2%)	21 (12.9%)

The median fever clearance time in the fully compliant patients for group CQ compared with CQ-MB was 8.0 and 8.8 hours respectively (p_WMW _= 0.725) and the median parasite clearance time was 91.3 and 86.4 hours respectively (p_WMW _= 0.140). The group comparisons are similar using the intention-to-treat approach.

### Acceptance of study drugs

The acceptance of the MB treatment was good, despite some blue staining of clothes by the children's urine. There was no response from 4/181 caretakers, 94/181 stated that they had no problem with the CQ-MB treatment, while 83/181 mentioned minor difficulties concerning the washing procedure of the stained clothes.

## Discussion

MB has been used systematically against malaria in different patient populations during the late 19^th ^and the early 20^th ^century, but the effects of this drug were poorly documented in these old studies [14,11,32]. The present report provides the first data on the safety and efficacy of methylene blue in young children with falciparum malaria in SSA. MB was chosen to be applied in combination with CQ for reasons of expected synergy and potential reversal of CQ resistance and because combination therapy is the new paradigm in malaria therapy [32,37,28]. Moreover, CQ has been the first-line treatment for malaria in Burkina Faso at the time of the study.

G6PD deficiency was diagnosed through a phenotypic method and this method was shown to have a good concordance with results from genotyping [23]. During treatment of 181 children, including 24 G6PD-deficient children with uncomplicated falciparum malaria in Burkina Faso, no drug related SAEs and, particularly, no cases of severe haemolysis were observed. This does not mean that SAEs can be excluded totally, but the likelihood is certainly smaller than the risk of young SSA children dying from malaria [34]. Moreover, no other adverse events likely to be related to the study drugs were noted. Thus, the findings from this study for the first time demonstrate the safety of a methylene blue-based combination in the treatment of malaria in young children of SSA. These results support previous findings on the safety of CQ-MB in G6PD sufficient adults in Europe and G6PD deficient adults in Burkina Faso [30,20]. Although there were no safety problems with the oral combination of CQ-MB, the administration of the bitter-tasting MB solution was sometimes difficult, especially in younger children. As a consequence, a paediatric formulation for taste-masking of MB is currently under development.

The findings from this study show that MB appears to be safe at an oral dose of up to 4 mg/kg/day over three days in SSA populations with dominating class III G6PD deficiency, despite its being on the list of drugs reported to potentially cause severe haemolysis in G6PD-deficient populations [12]. However, this listing at least in part, may have its origin in falsely attributing haemolysis caused by the underlying infectious disease to the drug used for treatment [3]. Nevertheless, as MB is a redox-cycling oxidant and G6PD has an important role in the elimination of reactive oxygen species in the erythrocyte, the safety of MB may be influenced by the prevailing type of G6PD deficiency [15]. Further studies are needed in populations where G6PD deficiency class II occurs [12].

The combination of dapsone and chloroproguanil (Lapdap) has recently been registered for malaria therapy in SSA [19]. With regard to the potential of haemolysis development in G6PD-deficient populations, MB belongs to the same risk category as dapsone [12]. Thus, our findings may also be reassuring regarding the safety of dapsone. Lapdap is currently undergoing phase IV studies in different SSA populations.

Compared to CQ resistance data from the surrounding villages, the observed rate of clinical failures during CQ treatment was surprisingly high in the urban/semi-urban population of this study (details will be published separately) [27]. In most of Burkina Faso, the level of resistance to CQ has remained remarkably stable during recent years [29,35,24]. However, much higher CQ failure rates were already documented from the capital town Ouagadougou [33]. As a consequence, a policy change regarding first-line treatment of uncomplicated malaria in Burkina Faso was decided upon in early 2005 and it is planned to use artemisinin-based combination therapy (ACT) starting in 2006.

There were no differences in baseline characteristics between the two study groups, with the exception of a higher haematocrit in the group CQ-MB compared to the CQ group, a finding likely to be explained by chance. The clinical failure rate was higher in the CQ arm (54%) compared to the CQ-MB arm (44%), but this difference was not significant. This result could possibly be explained by too low a dose of MB chosen in this study. Such an assumption is supported by much higher MB doses reported for treatment of malaria patients some 100 years ago [12,14,21,16]. In one of these studies, a good safety and efficacy of MB at oral doses of 20–50 mg/kg/day MB over several days to weeks was demonstrated during treatment of 40 young children with malaria in Brasil [11]. However, in these times treatment was usually uncontrolled, and efficacy outcomes in these old publications often were poorly documented. As a consequence of the lack of efficacy of the CQ-MB combination in this study, a MB dose finding study has been conducted in 2004 in the same study area. Findings confirm the efficacy of MB at higher doses and will be published separately.

In the present investigation, methylene blue was again well accepted by the study population [20]. Staining of clothes was not considered as a major problem by mothers. Only in a few cases, two to three traditional washes were needed before stains were totally removed (Sanon, unpublished data). This confirms results from a former experimental study on the reversibility of MB stains in local clothes and supports findings from an anthropological study – on community perceptions of blue urine and blue clothes – conducted during the rainy season of the year 2003 in the rural Nouna study area (Sanon, unpublished data).

In conclusion, MB has been shown to be safe for the treatment of uncomplicated falciparum malaria in young West African children with a high prevalence of G6PD deficiency. However, the efficacy of the CQ-MB combination has not been sufficient at the MB dose used in this study. Future studies need to assess the efficacy of MB at higher doses and in combination with appropriate partner drugs.

## Authors' contributions

P Meissner and G Mandi contributed equally to the study. P Meissner, G Mandi, S Witte, U Mansmann, A Jahn, I Walter-Sack, H Schirmer, B Kouyaté and O Müller designed the study. P Meissner, G Mandi, B Coulibaly, M Sanon, J Rengelshausen, W Schiek, G Mikus, J Burhenne and KD Riedel conducted the laboratory and clinical work. S Witte, U Mansmann and T Tapsoba did the statistical analysis. All authors contributed to the writing of the paper. O Müller was the principal investigator.
